# Hypothesis Testing of Inclusion of the Tolerance Interval for the Assessment of Food Safety

**DOI:** 10.1371/journal.pone.0141117

**Published:** 2015-10-28

**Authors:** Hungyen Chen, Hirohisa Kishino

**Affiliations:** 1 National Research Institute of Fisheries Science, Fisheries Research Agency, Kanagawa, 236–8648, Japan; 2 Graduate School of Agricultural and Life Sciences, The University of Tokyo, Tokyo, 113–8657, Japan; Huazhong University of Science and Technology, CHINA

## Abstract

In the testing of food quality and safety, we contrast the contents of the newly proposed food (genetically modified food) against those of conventional foods. Because the contents vary largely between crop varieties and production environments, we propose a two-sample test of substantial equivalence that examines the inclusion of the tolerance intervals of the two populations, the population of the contents of the proposed food, which we call the target population, and the population of the contents of the conventional food, which we call the reference population. Rejection of the test hypothesis guarantees that the contents of the proposed foods essentially do not include outliers in the population of the contents of the conventional food. The existing tolerance interval (TI_0_) is constructed to have at least a pre-specified level of the coverage probability. Here, we newly introduce the complementary tolerance interval (TI_1_) that is guaranteed to have at most a pre-specified level of the coverage probability. By applying TI_0_ and TI_1_ to the samples from the target population and the reference population respectively, we construct a test statistic for testing inclusion of the two tolerance intervals. To examine the performance of the testing procedure, we conducted a simulation that reflects the effects of gene and environment, and residual from a crop experiment. As a case study, we applied the hypothesis testing to test if the distribution of the protein content of rice in Kyushu area is included in the distribution of the protein content in the other areas in Japan.

## Introduction

The safety assessment of genetically modified (GM) foods was confirmed as an important issue in the Organization for Economic Cooperation and Development (OECD) discussion resumed in 1988. Substantial equivalence has been a starting point for the safety assessment for GM foods which is used worldwide since this approach was first suggested in 1993 [1]. Substantial equivalence embodies the concept that if a new food or food component is found to be substantially equivalent to an existing food or feed component, it can be treated in the same manner with respect to safety [2]. To decide if a modified product is substantially equivalent, the product is tested by the manufacturer for unexpected changes in a limited set of components such as toxins, nutrients, or allergens that are present in the unmodified food. Piaggio et al. [3] gave a clear framework of reporting of equivalence randomized trials. Ennis and Ennis [4,5] used an open interval to define equivalence and provided methods for testing a null hypothesis of nonequivalence. McNally et al. [6] proposed applying the generalized test function method in comparison to the confidence interval for assessing population bioequivalence. Herman and Price [7] examined research that has occurred over the past two decades relative to the mechanisms that affect crop composition in GM and traditionally bred crops.

In substantial equivalence tests of the population means, it is impossible to prove exact equality, so a buffer margin (*c*) for the treatment effect is defined. The equivalence is defined as the treatment effect being between *c* and −*c*.

$$\begin{array}{l} {\text{H}}_{0}\;:\left|\mu_{1}-\mu_{2}\right|\geq c\\ {\text{H}}_{1}\;:\left|\mu_{1}-\mu_{2}\right|<c \end{array}$$ (1)

**Fig 1:**
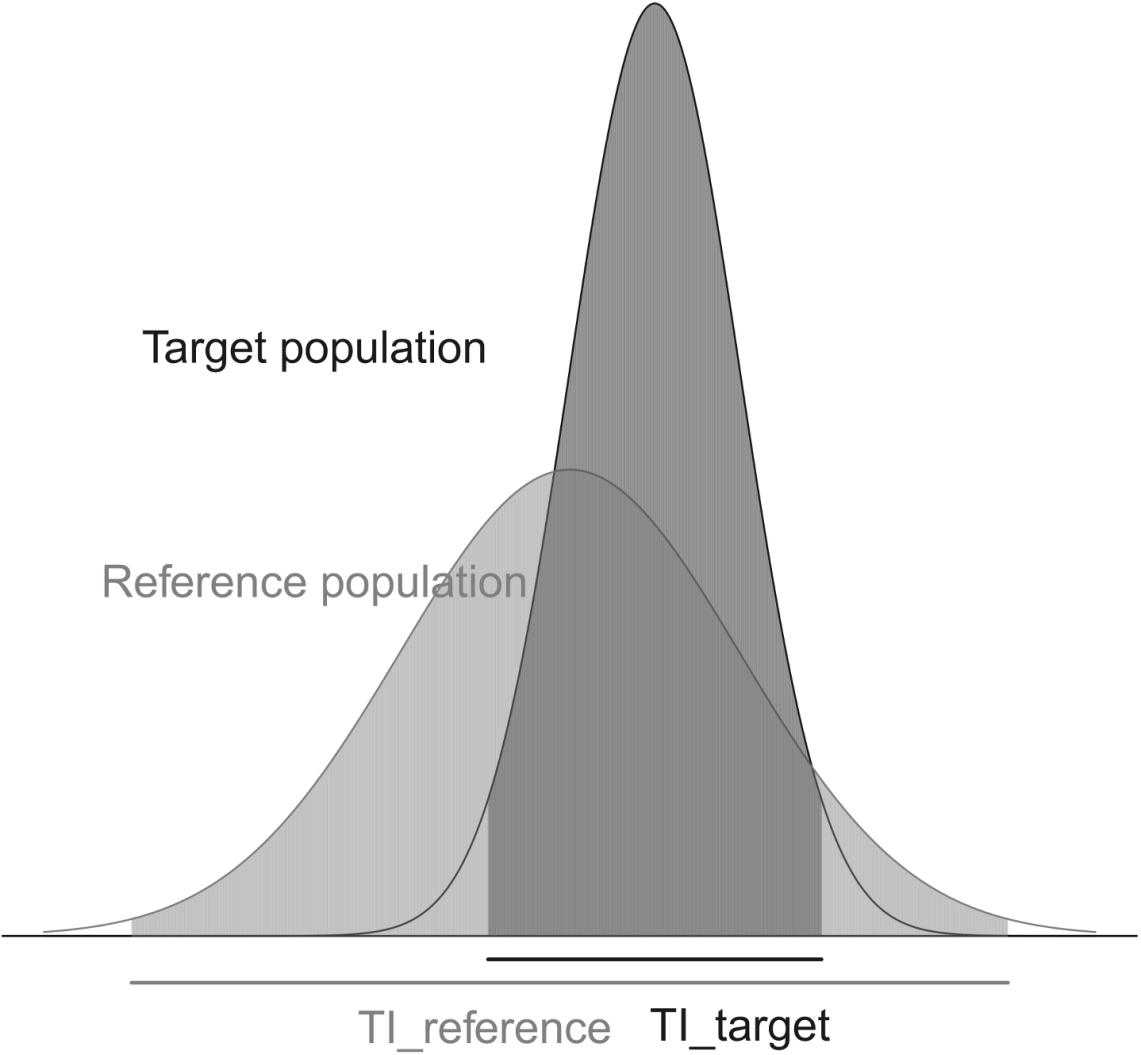
The distributions of two normal populations, the target population (POP_tar_) and the reference population (POP_ref_), and the tolerance intervals TI(*γ*_tar_, POP_tar_) and TI(*γ*_ref_, POP_ref_). As an example, *γ*_tar_ and *γ*_ref_ were set to 0.05 and 0.01 respectively.

A broad range of factors affect crop compositions, such as the genetic background [8,9], environmental factors [10,11], and agronomic practices [12,13]. Ricroch et al. [14] reviewed the published studies regarding the effect of genetic modification in comparison with the environmental and intervariety variations. Because the contents vary largely between crop varieties and production environments, the test of substantial equivalence should examine the inclusion of the tolerance intervals of the samples from the two populations, the population of the contents of the proposed food or feed, which we call the target population and denote as POP_tar_, and the population of the contents of the conventional food or feed, which we call the reference population and denote as POP_ref_ (Fig 1).

Statistical tolerance intervals are useful in practical applications in many areas and the construction of tolerance intervals has been extensively studied [15]. Formula for tolerance intervals (regions) for known and unknown mean and variance was given by Proschan [16] for univariate normal distribution and by Chew [17] for multivariate normal distribution. The tolerance interval procedure was developed for balanced one-way random model [18], general linear mixed models for balanced data [19] and unbalanced data [20]. A (1 − *γ*, 1 − *α*) tolerance interval (TI_0_) based on a sample is constructed so that it would include at least a proportion 1 − *γ* of the sampled population with confidence 1 − *α* [21]. Such a tolerance interval is usually referred to as (1 − *γ*)-content-(1 − *α*)-confidence (coverage) tolerance interval.

We introduced the complementary tolerance interval that is guaranteed to have at most a pre-specified level of the coverage probability. A (1 − *γ*, 1 − *α*) tolerance interval (TI_1_) based on a sample is constructed so that it would include at most a proportion 1 − *γ* of the sampled population with confidence 1 − *α*. By applying TI_0_ and TI_1_ to the samples from the target population and the reference population respectively, the rejection of the test guarantees that the target population essentially does not include outliers in the reference population.

## Material and Methods

### Two complementary tolerance intervals and two-sample hypothesis testing

We consider a sample *X* from a Gaussian population *N*(*μ*, *σ* ^2^). When the sample is collected by simple random sampling, the sample mean $\hat{\mu }$ follows *N*(*μ*, *σ* ^2^/*R*_0_), and the sample variance ${\hat{\sigma }}^{2}$ follows (*σ* ^2^/*m*) $\chi_{m}^{2}$. *R*_0_ is the sample size, and the degree of freedom, *m*, is *R*_0_ − 1. By allowing for the uncertainty of the sample mean and the sample variance, the conventional two-sided (1 − *γ*)-content, (1 − *α*)-confidence tolerance interval is defined as

$${\text{TI}}_{0}=\left(\hat{\mu }-\hat{\sigma }\sqrt{\frac{m\cdot \chi_{1:1-\gamma }^{2}(\text{ncp}=1/R_{0})}{\chi_{m;\alpha }^{2}(\text{ncp}=0)}},\hat{\mu }+\hat{\sigma }\sqrt{\frac{m\cdot \chi_{1:1-\gamma }^{2}(\text{ncp}=1/R_{0})}{\chi_{m;\alpha }^{2}(\text{ncp}=0)}}\right),$$ (2)

where $\chi_{1,1-\gamma ,1/R_{0}}^{2}$ denotes the upper 100(1 − *γ*)% point of the non-central chi-squared distribution with degree of freedom one and non-centrality parameter 1/*R*_0_, and $\chi_{m,\alpha }^{2}$ denotes the upper 100*α*% point of chi-squared distribution with degree of freedom *m* [22]. The notation ncp stands for non-centrality parameter. *R*_0_ is the ratio of *σ* ^2^ over the variance of $\hat{\mu }$, and *m* represents the ratio of 2${\left({\hat{\sigma }}^{2}\right)}^{2}$ over variance of *σ* ^2^. The tolerance interval TI_0_ covers at least 1 − *γ* of the population with the probability of 1 − *α*.

Here, we introduce a new tolerance interval TI_1_ defined by

$${\text{TI}}_{1}=\left(\hat{\mu }-\hat{\sigma }\sqrt{\frac{m\cdot \chi_{1:1-\gamma }^{2}(\text{ncp}=1/R_{0})}{\chi_{m;1-\alpha }^{2}(\text{ncp}=0)}},\hat{\mu }+\hat{\sigma }\sqrt{\frac{m\cdot \chi_{1:1-\gamma }^{2}(\text{ncp}=1/R_{0})}{\chi_{m;1-\alpha }^{2}(\text{ncp}=0)}}\right),$$ (3)

where $\chi_{m,1-\alpha }^{2}$ denotes the lower 100*α*% point of Chi-squared distribution with degree of freedom *m*. As is seen below, the tolerance interval TI_1_ covers at most 1 − *γ* of the population with the probability of 1 − *α*. By increasing the sample size, the two complementary tolerance intervals both converge to the population tolerance interval.

We contrast the tolerance interval of the target population, POP_tar_, with the tolerance interval of the reference population, POP_ref_. Given the values of *γ*_tar_ and *γ*_ref_ (*γ*_tar_ > *γ*_ref_), the null hypothesis is that the tolerance interval of POP_tar_ is not included in the tolerance interval of POP_ref_. The alternative is that the tolerance interval of POP_tar_ is included in the tolerance interval of POP_ref_. To make the dependence of the tolerance intervals on the sample X and population P explicit, we express them as TI_0_(*α*, *γ*, X), TI_1_(*α*, *γ*, X), and TI(*γ*, P). Our framework of testing substantial equivalence is to test the null hypothesis, H_0_, against the alternative hypothesis, H_1_.

$$\begin{array}{c} {\text{H}}_{0}\;:\;\text{TI}\left(\gamma_{\text{tar}},{\text{POP}}_{\text{tar}}\right)⊄\text{TI}\left(\gamma_{\text{ref}},{\text{POP}}_{\text{ref}}\right)\\ {\text{H}}_{1}\;:\;\text{TI}\left(\gamma_{\text{tar}},{\text{POP}}_{\text{tar}}\right)\subset \text{TI}\left(\gamma_{\text{ref}},{\text{POP}}_{\text{ref}}\right) \end{array}.$$ (4)

We define the test statistic as,

$$\begin{array}{l} \alpha_{\text{TI}01}=\alpha_{\text{TI}01}\left({\text{X}}_{\text{tar}},{\text{X}}_{\text{ref}}\right)\\ \;=\text{arg min}\left\{\alpha |\;{\text{TI}}_{0}\left(\alpha /2,\gamma_{\text{tar}},{\text{X}}_{\text{tar}}\right)\subset {\text{TI}}_{1}\left(\alpha /2,\gamma_{\text{ref}},{\text{X}}_{\text{ref}}\right)\right\} \end{array},$$ (5)

where X_tar_ and X_ref_ are the sample from POP_tar_ and POP_ref_ respectively. The p value is obtained by locating the test statistic on its distribution for the case of TI(*γ*_tar_, POP_tar_) = TI(*γ*_ref_, POP_ref_).

### Mixed effect model and the coverage probabilities of the tolerance intervals

The two complementary tolerance intervals (Eqs (2) and (3)) can be generalized for the non-iid samples. The effective sample size, *R*_0_, is obtained by comparing the variance of the estimated global mean with the total variance: $V\left[\hat{\mu }\right]=\left({\hat{\sigma }}_{T}^{2}\right)/R_{0}$. The effective degree of freedom, *m*, is obtained by equating the estimated variance of the estimated total variance and the expected variance of the estimated total variance by the Satterthwaite’s chi-square approximation: $V\left[{\hat{\sigma }}_{T}^{2}\right]\approx 2{\left({\hat{\sigma }}_{T}^{2}\right)}^{2}/m$.

As an example, we consider the hypothetical samples with random genetic and environmental effects. The hypothetical samples reflect the maize samples of 61 lines from eight multi-site field studies. The field sites represented 47 unique environments in the commercial maize-growing regions of the United States, Canada, Chile and Argentina [23]. The experimental design used at each field site was a randomized complete block design containing three of four blocks. Variances of random components of concentrations of two analytes (tryptophan and oleic acid) were used to generate the simulated data. Table 1 shows the variances of random components of tryptophan and oleic acid. The variance component of environmental effect is large for tryptophan, whereas the genetic effect is the major component for oleic acid.

**Table 1:** Variance of random components of a maize experiment.

	% Total variance				
Analyte	G	E	GxE	B	*ε*
Tryptophan	6.7	71.6	3.5	0.1	18.1
Oleic acid	55.6	16.4	8.7	0.1	19.3

G, genotype; E, environment; GxE, interaction of genotype and environment; B, block; *ε*, residual.

Table 2 shows the simulation setting with total number of environment, n_E_ = 50, total number of genotype, n_G_ = 50 and number of blocks per environment, n_B_ = 4. We generated 1,000 sample datasets by normal random numbers with the variances in Table 1. We applied a linear mixed model to each of the dataset, and estimated the total mean and the variance components. The variance of total mean and the total variance, which are required for the calculation of *R*_0_ and *m*, were estimated by the variance among the 100 runs of parametric bootstrap.

**Table 2:** Design setup for the simulation study.

	Genotype				
Environment	G01−G10	G11−G20	G21−G30	G31−G40	G41−G50
E01−E10	√	√			
E11−E20		√	√		
E21−E30			√	√	
E31−E40				√	√
E41−E50	√				√

**Fig 2:**
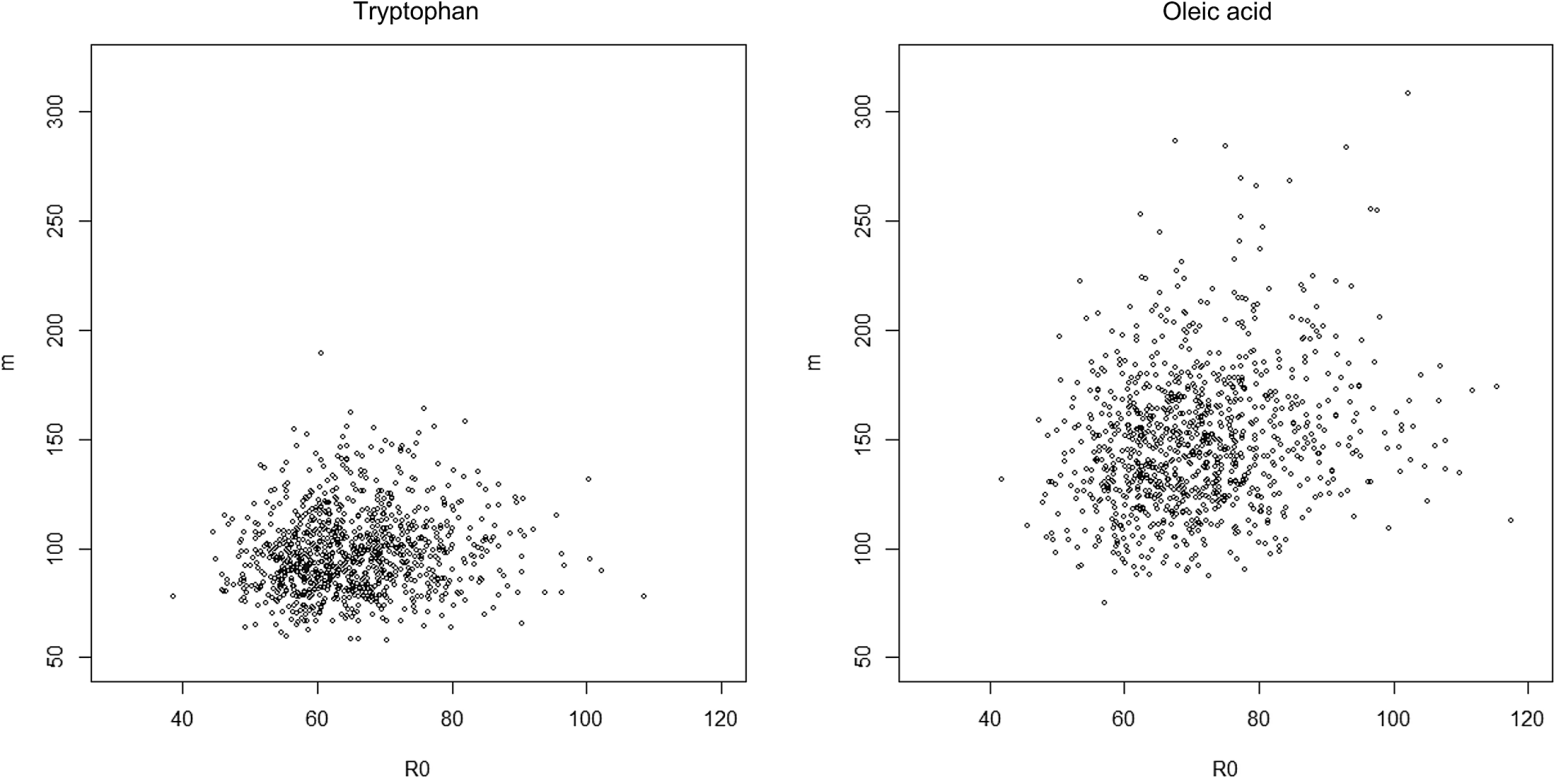
The distributions of the estimated effective sample size (*R*_0_) and the effective degree of freedom (*m*) for tryptophan and oleic acid separately.

**Fig 3:**
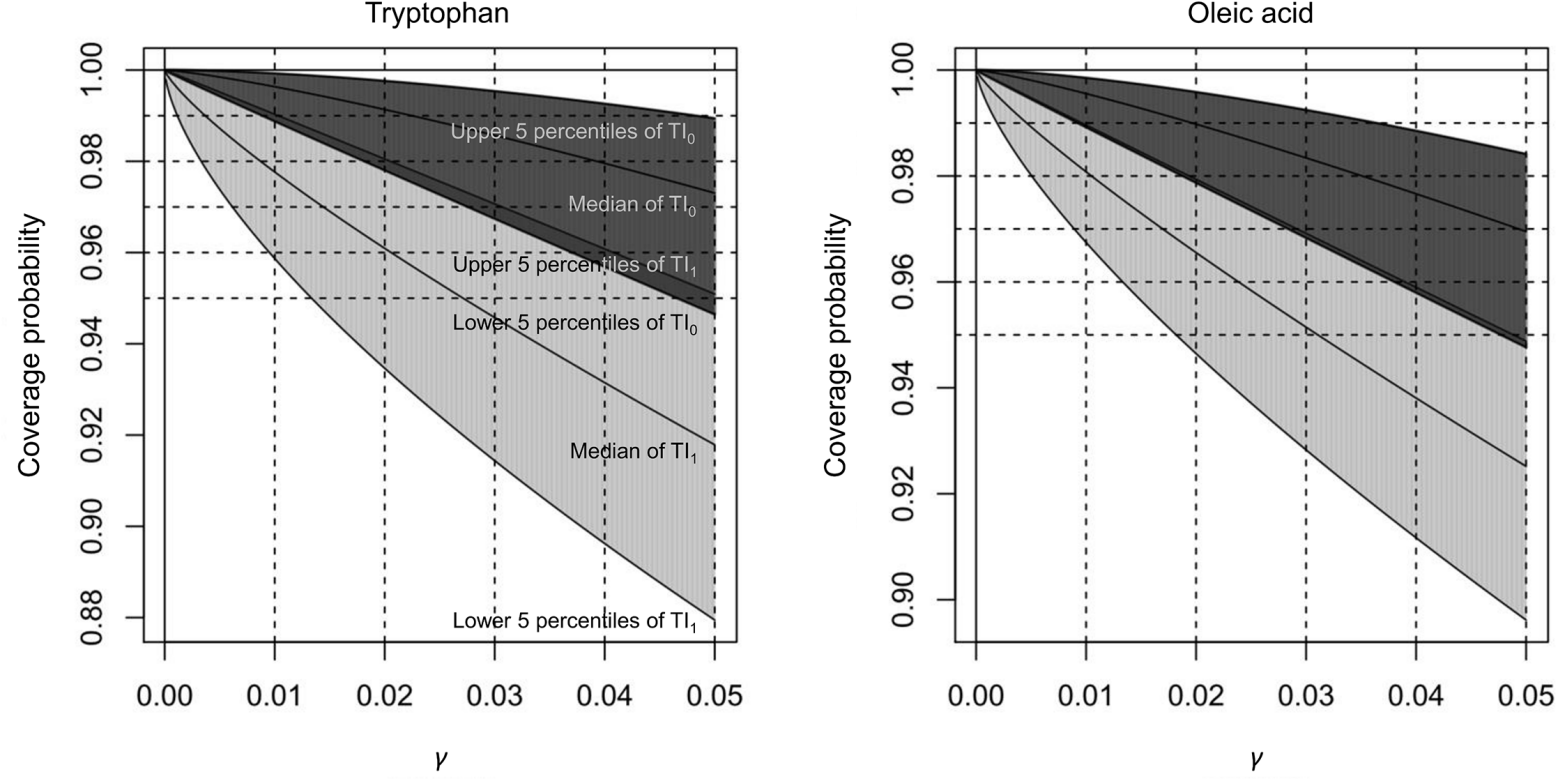
The median, lower and upper 5 percentiles of the coverage probabilities of the tolerance intervals, TI_0_ (dark gray area) and TI_1_ (light gray area). For each area, middle, lower, and upper curves represent the median, lower and upper 5 percentiles of the coverage probabilities respectively. The 5 dotted horizontal lines represent the nominal coverage probability (1 –*γ*) for the 5 marked *γ* (0.01, 0.02, 0.03, 0.04, and 0.05).

The estimated *m* and *R*_0_ were distributed widely (Fig 2). The means of the estimated *m* were 98.0 for tryptophan and 149.3 for oleic acid. The means of the estimated *R*_0_ were 65.4 for tryptophan and 70.8 for oleic acid. Fig 3 shows the median, lower and upper 5 percentiles of the coverage probabilities of the tolerance intervals, TI_0_ and TI_1_. The coverage probability of TI_0_ is larger than the nominal coverage probability (1 –γ) with probability 0.95. For the value of *γ* = 0.01 (see the first dotted vertical line from the left on both panels), with probability 95%, the lower 5 percentiles of coverage probabilities of TI_0_ were larger than 98.9% and 98.9% for tryptophan and oleic acid respectively; the upper 5 percentiles of coverage probabilities of TI_0_ were smaller than 99.9% and 99.8% for tryptophan and oleic acid respectively; the medians were 99.6% for both tryptophan and oleic acid. This means that TI_0_ covers at least 1 − *γ* of the population with the probability 95%.

On the other hand, the coverage probability of TI_1_ is smaller than the nominal coverage probability (1 –γ) with probability 0.95. For the value of *γ* = 0.01 (see the first dotted vertical line form the left on both panels), with probability 95%, the upper 5 percentiles of coverage probabilities of TI_1_ were smaller than 99.0% for both tryptophan and oleic acid; the lower 5 percentiles of coverage probabilities of TI_1_ were larger than 95.9% and 96.7% for tryptophan and oleic acid respectively; the medians were 97.8% and 98.1% for tryptophan and oleic acid respectively. This means that TI_1_ covers at most 1 − *γ* of the population with the probability 95%.

## Results

### The p values and the power of the hypothesis test: a simulation study

To investigate the performance of the test procedure, we conducted a simulation study of contrasting two normal populations. The value of *γ*_tar_ and *γ*_ref_ were set to 0.05 and 0.01 respectively. The POP_tar_ and POP_ref_ are assumed to follow normal distribution with means *μ*_tar_ = *μ*_ref_ = 0. By solving the relation TI(*γ*_tar_, POP_tar_) = TI(*γ*_ref_, POP_ref_), we obtained *σ*_tar0_ = 1.41*σ*_ref0_ as the population parameter of the null hypothesis. The sample sizes were fixed to 50 for both POP_tar_ and POP_ref_. The distribution under the null hypothesis was obtained by 10,000 simulation trials. For each value of *σ*_tar_ = (1 − 0.05*i*)*σ*_tar0_, *i* = 0, 1, 2, …, 10, 1,000 values of *α*_TI01_ were generated randomly.

**Fig 4:**
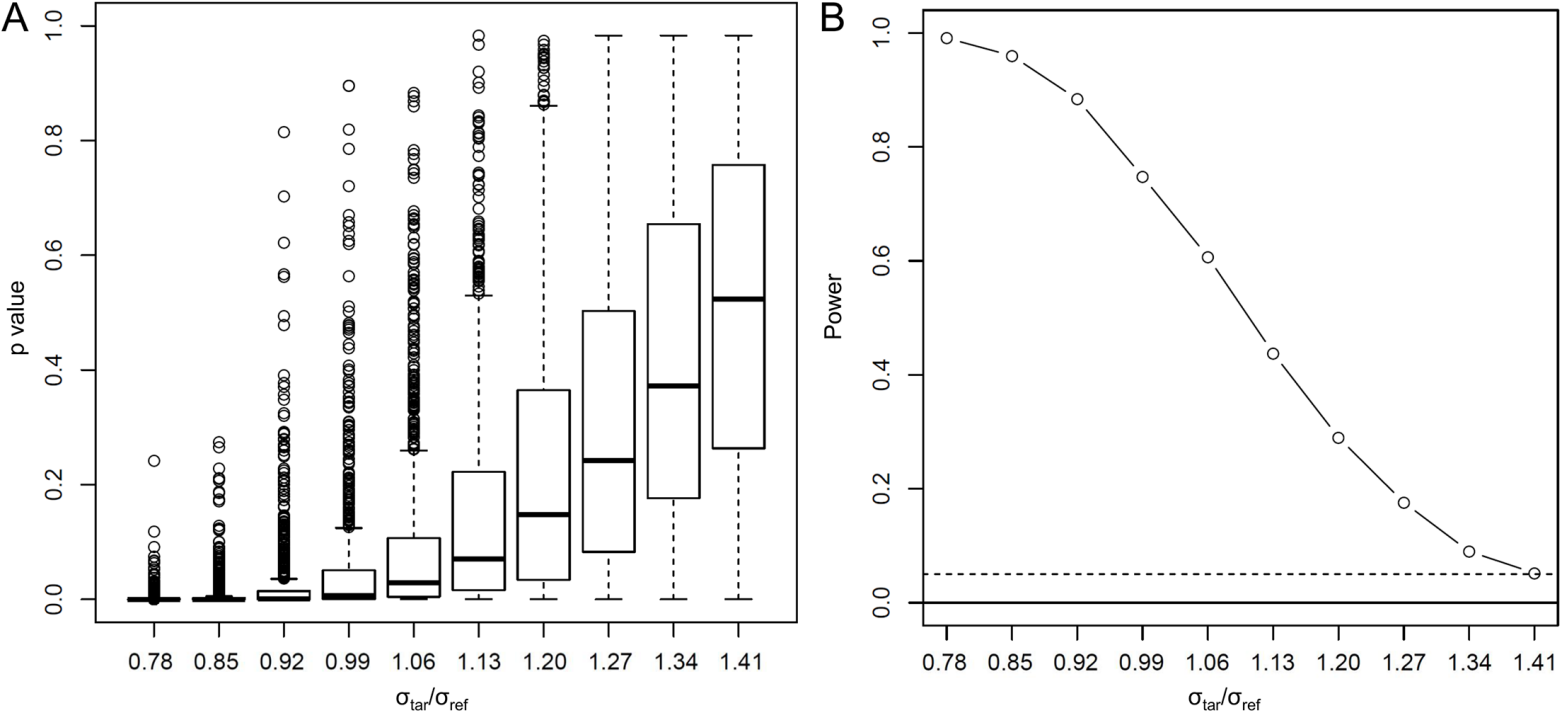
The p value (A) and the power (B) of the test with sample size 50 for both target population and reference populations. See Material and methods for the definition of the defined test statistics *α*_TI01_. The dotted line represents the significance level of 0.05.

Fig 4A shows the distribution of the p-values and the power of the test with the significance level of 0.05. The p-value followed mostly the uniform distribution when the null hypothesis is real (*σ*_tar_ /*σ*_ref_ = 1.41). The power at the null hypothesis was 0.051, which was slightly larger but close to the significance level of 0.05 (Fig 4B). The power increased to 0.606 for the case of *σ*_tar_ /*σ*_ref_ = 1.06, and to 0.999 for the case of *σ*_tar_ /*σ*_ref_ = 0.78.

To see the effect of sample size, we conducted the simulation for the cases of sample sizes to 100, 150 and 200 for both POP_tar_ and POP_ref_. The power to reject the null hypothesis with the significance level of 0.05 is shown in Table 3. The power for the case of *σ*_tar_ /*σ*_ref_ = 1.06 became 0.852 when the sample size was doubled, and 0.987 when the sample size was 200. On the other hand, the power for the case of *σ*_tar_ /*σ*_ref_ = 1.41 stayed nearly at the value of 0.05.

**Table 3:** The power to reject the null hypothesis with significance level of 0.05 for each combination of sample size and the size of *σ*_tar_/*σ*_ref_.

Sample size	*σ*_tar_/*σ*_ref_					
	1.06	1.13	1.20	1.27	1.34	1.41
50	0.606	0.449	0.287	0.171	0.097	0.051
100	0.852	0.684	0.456	0.246	0.114	0.053
150	0.948	0.816	0.578	0.343	0.164	0.045
200	0.987	0.915	0.708	0.390	0.183	0.058

### A case study of testing inclusion of tolerance intervals: Contrasting protein composition of rice in Kyushu against other areas in Japan

As an example of empirical study, we applied the hypothesis testing to test if the protein value of rice in Kyushu area (Kyushu, including prefectures Fukuoka and Kagoshima) was included in the other areas in Japan (Japan). We downloaded the rice component data for Japan from The Food Composition Database for Safety Assessment of Genetically Modified Crops as Foods and Feeds [24,25]. It is third-party data and not owned by the authors. Major varieties of non-glutinous rice cultivated and distributed in Japan were collected from 1999 to 2009 (except for 2003 and 2004). A total of 15 or 16 samples consisting of 10−12 varieties were collected every year. The production areas are located in Japan stretching from the far north to south of the country. Table 4 shows the number of samples of different varieties and production areas. In total, the sample X_Japan_ includes 120 rice samples of varieties and the sample X_Kyushu_ includes 18 rice samples of varieties.

**Table 4:** Number of rice sample of varieties and production areas.

Production area	Variety														
(Prefecture)	Aic	Aki	Don	Hae	Han	Hin	Hit	Hos	Kin	Kir	Kos	Mas	Tsu	Yum	Total
Aichi	5														5
Akita		9													9
Aomori												2	4	2	8
Fukui					9						1				10
Fukuoka						9									9
Hokkaido								9		9					18
Hyogo			1												1
Ibaraki											8				8
Iwate							9								9
Kagoshima						9									9
Miyagi							9								9
Nagano		2													2
Niigata			4								9				13
Shiga									9						9
Tochigi											9				9
Yamagata		1		9											10
Total	5	12	5	9	9	18	18	9	9	9	27	2	4	2	138

Aic, Aichinokaori; Aki, Akitakomachi; Don, Dontokoi; Hae, Haenuki; Han, Hanaechizen; Hin, Hinohikari; Hit, Hitomebore; Hos, Hoshinoyume; Kin, Kinuhikari; Kir, Kirara397; Kos, Koshihikari; Mas, Masshigura; Tsu, Tsugaruroman; Yum, Yumeakar.

To test if the protein value of rice in Kyushu was included in Japan, we applied TI_0_ and TI_1_ to the samples from Kyushu and Japan respectively. The null hypothesis is that the tolerance interval of the protein of rice in Kyushu was not included in the tolerance interval of that in Japan. The alternative is that the tolerance interval of the protein of rice in Kyushu was included in the tolerance interval of that in Japan. The value of *γ*_Kyushu_ and *γ*_Japan_ were set to 0.05 and 0.01 respectively.

Using a linear mixed-effect model we estimated the total mean of POP_Japan_ as *μ*_Japan_ = 6.60 and random effects $\sigma_{\text{G},\text{Japan}}^{2},\;\sigma_{\text{E},\text{Japan}}^{2}$ and $\sigma_{\text{GxE},\text{Japan}}^{2}$ = 0.05, 0.07 and 0 respectively, and the error term, $\sigma_{\varepsilon ,\text{Japan}}^{2}$ = 0.19. The total variance $\sigma_{\text{T},\text{Japan}}^{2}$ = 0.31. The variance of the estimated total mean and that of the estimated total variance were estimated as $V\left[{\hat{\mu }}_{\text{Japan}}\right]=0.0128$ and $V\left[{\hat{\sigma }}_{\text{T},\text{Japan}}^{2}\right]=0.00365$ respectively by 1,000 runs of parametric bootstrap. These values provide the effective sample size, $R_{0,\text{Japan}}={\hat{\sigma }}_{\text{T},\text{Japan}}^{2}/V\left[{\hat{\mu }}_{\text{Japan}}\right]=24.28$ and the effective degree of freedom, $m=2{\left({\hat{\sigma }}_{\text{T,Japan}}^{2}\right)}^{2}/V\left[{\hat{\sigma }}_{\text{T,Japan}}^{2}\right]=52.70$. The sample from POP_Kyushu_ is assumed to be an iid sample from normal distribution with mean *μ*_Kyushu_ = 6.92 and variance $\sigma_{\text{T},\text{Kyushu}}^{2}$ = 0.22. In this case, *R*_0,Kyushu_ is the sample size, 18, and *m*_Kyushu_ is *R*_0,Kyushu_ − 1 = 17. With these values of *R*_0_’s and *m*’s, we obtained TI_1_(*α* = 0.05, *γ* = 0.01, X_Japan_) as (5.34, 7.86) and TI_0_(*α* = 0.05, *γ* = 0.05, X_Kyushu_) as (5.59, 8.24). The latter does not include the former.

**Fig 5:**
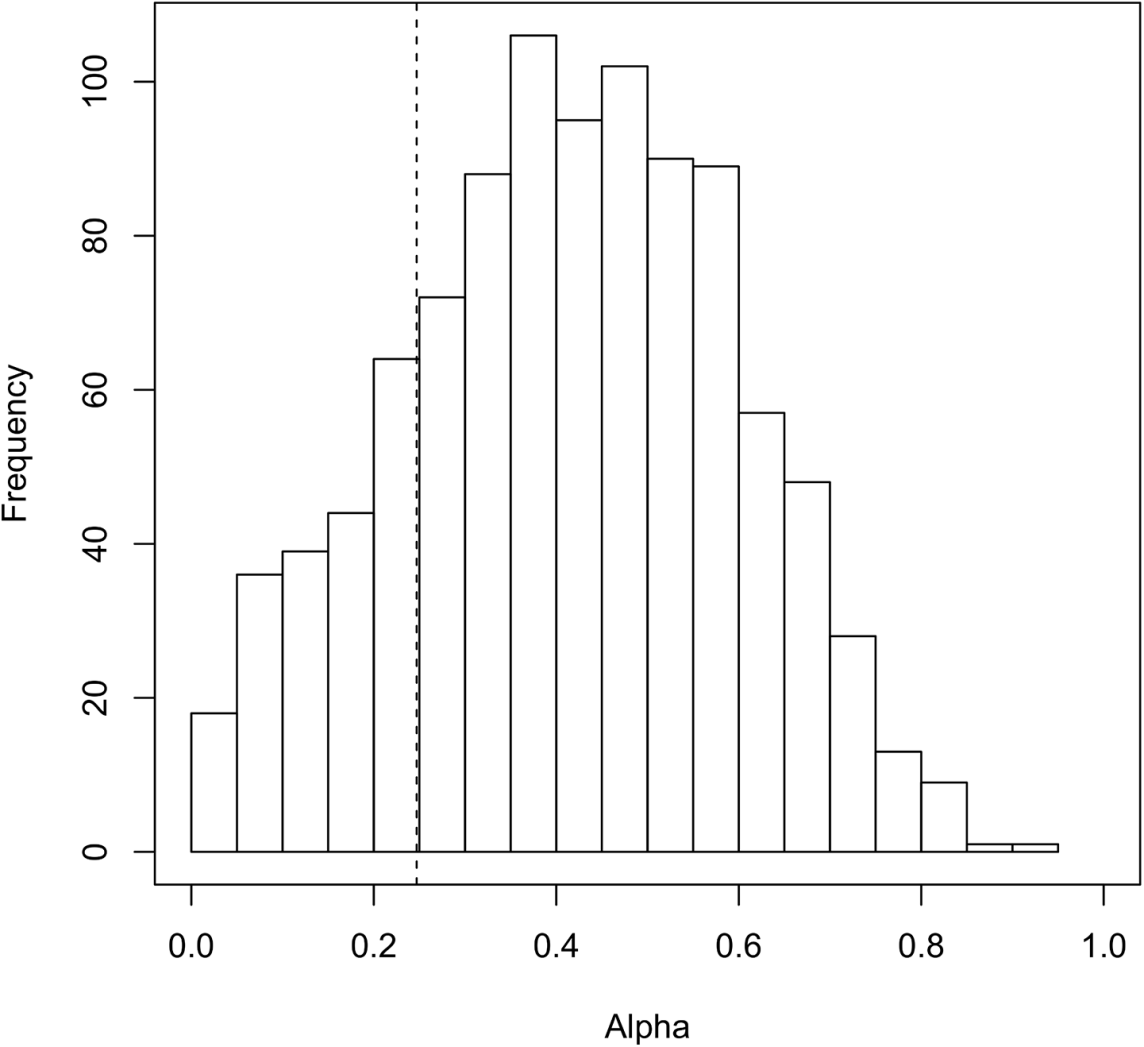
Distribution of *α*_TI01_ obtained by 1,000 simulation trials under the null hypothesis. The dotted line represents the value of 0.247.

The value of the test statistic, *α*_TI01_, was numerically obtained as 0.247 by solving the Eq (3). We obtained the p value by locating the value of *α*_TI01_ on the distribution under the null hypothesis. We generated this distribution by parametric bootstrap, assuming *μ*_Kyushu_ = *μ*_Japan_ and *σ*_T,Kyushu_ = 1.41*σ*_T,Japan_. Without losing generosity, we assumed *μ*_Kyushu_ = *μ*_Japan_ = 0 and *σ*_T,Japan_ = 1. The iid sample of Kyushu was generated by normal random numbers with mean 0 and standard deviation 1.41. As for the sample of Japan, we generated the genetic effect (G), environmental effect (E), the G×E interaction and the error term, by decomposing the total variance into the variance components by the relative size of the estimated variance components. We generated 1,000 sets of the data. For each of the simulated data, we estimated the means and the total variances of Kyushu and Japan. We estimated their variances by 100 parametric bootstrap. With these estimates, we obtained *R*_0_’s and *m*’s, and the value of *α*_TI01_. From 1,000 values of *α*_TI01_, we obtained the cumulative distribution of *α*_TI01_ under the null hypothesis (Fig 5). As a result, we obtained the p value as 0.195.

## Conclusion

In this study, we proposed a hypothesis test of inclusion of tolerance interval using the existing tolerance interval and a newly introduced the complementary tolerance interval. The result of simulation showed that the power of the test for the case of *σ*_tar_ /*σ*_ref_ = 1.41 stayed nearly at the value of 0.05 (Fig 4), which means that the testing procedure is almost unbiased. However, the test statistic, *α*_TI01_, is complex in form, and we could not attach a direct interpretation to it. We need make further effort to develop candidates of test statistics that measure the extent of coverage or non-coverage of the target population by the reference population. The mixed effect model enables unbiased estimation of the effective sample size and the effective degree of freedom, when the samples consist of subsamples collected in various conditions of genetic factors and environmental factors. However, a survey may be designed to collect the samples of matched controls. Another promising project is to develop a testing procedure for such samples.

As an alternative to the testing non-inferiority or substantial equivalence of the population mean, the proposed test examines the “range” of the distribution. A statistical test on the range of the distribution may be useful, especially when it is difficult to formulate the distribution by a simple statistical model. If a large sample is available, it is possible to construct non-parametric tolerance intervals [26,27]. The future study will investigate the statistical property of the non-parametric testing procedure.
